# Is the risk of Alzheimer’s disease and dementia declining?

**DOI:** 10.1186/s13195-015-0118-1

**Published:** 2015-03-26

**Authors:** Kenneth M Langa

**Affiliations:** Division of General Medicine, University of Michigan Health System, 2800 Plymouth Road, Bldg 16, Room 430 W, Ann Arbor, MI 48109-0429 USA; Veterans Affairs Center for Practice Management and Outcomes Research, 2215 Fuller Road, Ann Arbor, MI 48105 USA; Institute for Social Research, University of Michigan, 426 Thompson Street, Ann Arbor, MI 48105 USA; Institute of Gerontology, University of Michigan, 300 N. Ingalls Street, Ann Arbor, MI 48109 USA; Institute for Healthcare Policy and Innovation, University of Michigan, 2800 Plymouth Road, Ann Arbor, MI 48109 USA

## Abstract

**Background:**

The number of older adults with dementia will increase around the world in the decades ahead as populations age. Current estimates suggest that about 4.2 million adults in the US have dementia and that the attributable economic cost of their care is about $200 billion per year. The worldwide dementia prevalence is estimated at 44.3 million people and the total cost at $604 billion per year. It is expected that the worldwide prevalence will triple to 135.5 million by 2050. However, a number of recent population-based studies from countries around the world suggest that the age-specific risk of dementia may be declining, which could help moderate the expected increase in dementia cases that will accompany the growing number of older adults.

**Discussion:**

At least nine recent population-based studies of dementia incidence or prevalence have shown a declining age-specific risk in the US, England, The Netherlands, Sweden, and Denmark. A number of factors, especially rising levels of education and more aggressive treatment of key cardiovascular risk factors such as hypertension and hypercholesterolemia, may be leading to improving ‘brain health’ and declining age-specific risk of Alzheimer’s disease and dementia in countries around the world.

**Summary:**

Multiple epidemiological studies from around the world suggest an optimistic trend of declining population dementia risk in high-income countries over the past 25 years. Rising levels of education and more widespread and successful treatment of key cardiovascular risk factors may be the driving factors accounting for this decline in dementia risk. Whether this optimistic trend will continue in the face of rising worldwide levels of obesity and diabetes and whether this trend is also occurring in low- and middle-income countries are key unanswered questions which will have enormous implications for the extent of the future worldwide impact of Alzheimer’s disease and dementia on patients, families, and societies in the decades ahead.

Dementia, a decline in memory and other cognitive functions severe enough to cause disability in daily activities, has a large and growing impact on older adults, their families, and government programs in the US and around the world. In 2010, about 4.2 million adults in the US and more than 135 million around the world had dementia [1]. The economic impact of dementia, including a large burden of unpaid caregiving provided by families, is estimated at $200 billion per year in the US [2] and $600 billion worldwide [3], which is greater than the economic impact of important and common chronic diseases, such as heart disease and cancer. Because the incidence of dementia rises sharply over the age of 75, the estimated growth in the worldwide elderly population in the decades ahead (from about 600 million now to 1.5 billion in 2050) is expected to lead to a tripling of dementia cases by 2050 unless new interventions prevent or slow the trajectory of cognitive decline [1].

Owing to this large and growing impact of dementia, governments around the world have made a priority of expanding the collection of data on individuals and populations to better understand, address, and track the current and future impact of the dementia epidemic. For instance, President Obama signed the National Alzheimer’s Project Act into law in 2011, directing new US government efforts for improving treatments and prevention and collecting data to track progress of these efforts over time. The G8 Dementia Summit was held in London in 2013 in recognition of the growing global impact of Alzheimer’s disease (AD) and dementia and to begin to coordinate efforts for international collaboration and data sharing. Finally, the World Health Organization recently declared dementia to be a ‘public health priority’ which should be on the public health agenda of all countries.

Although the large growth in the number of older adults in the coming decades will lead to an increase in dementia cases in countries around the world, a number of recent studies have suggested that the age-specific risk of dementia has actually decreased in high-income countries over the last 25 years, possibly due to increasing levels of education and more aggressive treatment of cardiovascular risk factors that increase the risk of cognitive decline (for example, hypertension, hypercholesterolemia, and diabetes) [4]. However, it is unclear whether this optimistic trend in high-income countries will continue in the face of rising levels of obesity and diabetes, and it is also unclear whether there has been a similar or opposite trend in low- and middle-income countries [5,6].

## Recent trends important to brain health

Over the last 25 years, many countries have seen increases in obesity, diabetes, and hypertension, all of which have been linked to an increase in dementia risk. However, at the same time, there have been important changes in treatments for these cardiovascular risk factors, including more widespread and intensive medication treatments. For instance, in the US, achievement of blood glucose targets for diabetes has increased from 51% in 1990 to 58% in 2010, and adequate control of blood pressure (from 27% of those with diagnosed hypertension in 1990 to 50% in 2008) and high cholesterol (9% in 1999 to 27% in 2008) has also increased significantly [7-9]. These improvements in cardiovascular risk factor control have helped cut the key vascular-related complications of diabetes—myocardial infarction, stroke, and amputations—in half over the last 25 years [9] and by extension have likely had important ‘spillover’ benefits for brain health and the risk for cognitive decline and dementia.

A recent study used current estimates of the proportion of AD cases that are due to various cardiovascular risk factors (that is, the population-attributable risk for these risk factors), including hypertension, diabetes, and physical inactivity, to identify the proportion of cases that could theoretically be avoided by better prevention or control of these risk factors [10]. The study estimated that about 3% of worldwide cases of AD are due to diabetes, 5% to hypertension, and 13% to physical inactivity, suggesting that better prevention and control of these risk factors, while certainly not eliminating the majority of dementia cases, could help prevent millions of cases worldwide in the decades ahead.

A number of ongoing randomized clinical trials are directly testing whether lifestyle interventions to improve the vascular risk profiles of middle-aged and older adults will decrease their risk for cognitive decline and dementia. The Lifestyle Interventions and Independence for Elders (LIFE) Study tested whether a structured moderate-intensity physical-activity program would decrease the risk for mobility limitations and cognitive decline in adults who are 70 to 89 years old [11]. The LIFE intervention had a significant effect on decreasing mobility limitations, but the trial results for cognitive outcomes have not yet been reported. The Finnish Geriatric Intervention Study to Prevent Cognitive Impairment and Disability (FINGER) is randomly assigning adults who are 60 to 77 years old to a multi-pronged intervention that included diet advice, physical exercise, cognitive training, social activities, and management of cardiovascular risk factors, while the control group received only standard health advice [12]. The preliminary results from FINGER were reported at the Alzheimer’s Association International Conference in 2014 and showed that, after 2 years of follow-up, individuals randomly assigned to the multi-pronged lifestyle intervention had significantly better cognitive outcomes than the control group. The final results from FINGER have yet to be published.

The other important worldwide trend relevant to brain health over the last 25 years has been the large growth in educational attainment in both developed and developing countries. The proportion of 30- to 34-year-olds with a college education has increased in wealthy countries from 12% in 1970 to 27% in 2010 and from 3% to 11% in low- and middle-income countries [13]. A major tenet of the ‘cognitive reserve’ hypothesis is that education facilitates the development of compensatory neural circuits that provide an increased capacity to withstand damage from vascular and inflammatory brain insults, thereby delaying dementia onset and compressing cognitive morbidity closer to the end of life [14]. Therefore, rising levels of education may be contributing to a decreasing risk for AD and dementia among individuals around the world by increasing the stock of cognitive reserve among those who have been able to take advantage of these expanding educational opportunities. Of course, it should be noted that increased levels of education likely have an impact on later-life brain health through multiple and complex pathways, not just a direct effect on brain biology and cognitive reserve. For instance, more education is associated with higher levels of wealth, occupations that lead to more cognitive stimulation during one’s working life, and health behaviors (for example, lower rates of smoking and obesity and higher rates of physical activity) that confer increased protection against cognitive and physical decline [15].

## Recent studies of trends in dementia incidence and prevalence

A growing number of studies, at least nine over the past 10 years, have shown a declining risk for dementia incidence or prevalence in high-income countries, including the US, England, The Netherlands, Sweden, and Denmark. The Rotterdam Study, an ongoing population-based prospective cohort study, has provided the most direct evidence that improved cardiovascular risk factor control may be leading to healthier brains. Dementia incidence decreased in cohorts of older adults in Rotterdam between 1990 and 2010, and brain magnetic resonance imaging (MRI) performed in more recent cohorts shows significantly less brain atrophy and fewer vascular-related brain lesions compared with MRI performed in the earlier cohorts [16].

Recent findings from the UK Cognitive Function and Ageing Study (CFAS) provide additional support for a declining risk of dementia in older adults over the last 20 years [17]. CFAS studied adults 65 or older in Cambridgeshire, Newcastle, and Nottingham in the early 1990s (CFAS I) and again around 2010 (CFAS II). Importantly, the same standardized algorithmic diagnostic process was used for both time periods in order to avoid diagnostic drift and to maximize the validity of the prevalence comparisons across the two time periods. Between 1991 and 2011, the age- and sex-standardized dementia prevalence declined from 8.3% to 6.5% in these regions in England. This decline translated to a 24% decrease in the expected number of dementia cases in England in 2011. The CFAS authors hypothesized that increased education levels and better primary prevention of cardiovascular risk factors have likely contributed to the declining risk for dementia in England over the past 25 years.

An important methodological issue to consider in drawing conclusions from the CFAS findings, as well as findings from other studies of trends in dementia prevalence, consists of potential changes in study response rate over time. The response rate for the CFAS I field work was 80%, whereas the response rate for CFAS II was 56%. If individuals with dementia were more likely to be excluded from CFAS II, this could lead to a falsely low dementia prevalence rate. However, the CFAS investigators performed sensitivity analyses to address whether the drop in response rates could explain all of the decline in dementia prevalence, and still found evidence for significant decline even assuming a high likelihood of dementia in those who refused to participate in CFAS II. Nonetheless, with response rates for many population-based surveys declining over the last decades, it is important to consider and address this key methodological issue when drawing conclusions about trends in dementia and other chronic diseases.

## Conclusions and future uncertainties

Worldwide dementia cases will likely grow significantly over the next 40 years because of increasing life expectancies and the aging of populations worldwide. However, a growing number of studies now suggest a declining age-specific risk of dementia in high-income countries over the last 25 years, which, if continued, could help moderate the future worldwide growth in dementia cases. Rising levels of education and more widespread and successful treatment of key cardiovascular risk factors may be the driving factors accounting for this decline in dementia risk. Whether this optimistic trend will continue in the face of rising worldwide levels of obesity and diabetes and whether this trend is also occurring in low- and middle-income countries are key unanswered questions which will have enormous implications for the extent of the future worldwide impact of AD and dementia on patients, families, and societies in the decades ahead. Future studies that track the prevalence and costs of dementia in countries around the world will be extremely important as populations age worldwide, both to better understand the full societal impact of dementia and to help identify key medical and socioeconomic factors that should be targeted by interventions to decrease dementia risk.

## Abbreviations

AD: Alzheimer’s disease
CFAS: Cognitive function and ageing study
FINGER: Finnish geriatric intervention study to prevent cognitive impairment and disability
LIFE: Lifestyle interventions and independence for elders
MRI: Magnetic resonance imaging
